# An “orientation sphere” visualization for examining animal head movements

**DOI:** 10.1002/ece3.6197

**Published:** 2020-03-24

**Authors:** Rory P. Wilson, Hannah J. Williams, Mark D. Holton, Agustina di Virgilio, Luca Börger, Jonathan R. Potts, Richard Gunner, Alex Arkwright, Andreas Fahlman, Nigel C. Bennett, Abdulaziz Alagaili, Nik C. Cole, Carlos M. Duarte, David M. Scantlebury

**Affiliations:** ^1^ Department of Biosciences College of Science Swansea University Swansea UK; ^2^ Department of Computing Science College of Science Swansea University Swansea UK; ^3^ Grupo de Biología de la Conservación Laboratorio Ecotono INIBIOMA (CONICET‐Universidad Nacional del Comahue) Bariloche Argentina; ^4^ Grupo de Ecología Cuantitativa INIBIOMA (CONICET‐Universidad Nacional del Comahue) Bariloche Argentina; ^5^ School of Mathematics and Statistics University of Sheffield Sheffield UK; ^6^ Fundación Oceanogràfic de la Comunitat Valenciana Valencia Spain; ^7^ Department of Zoology and Entomology Mammal Research Institute University of Pretoria Pretoria South Africa; ^8^ Zoology Department King Saud University Riyadh Saudi Arabia; ^9^ Mauritian Wildlife Foundation Vacoas Mauritius; ^10^ Durrell Wildlife Conservation Trust Jersey Channel Islands; ^11^ Red Sea Research Centre King Abdullah University of Science and Technology Thuwal Saudi Arabia; ^12^ School of Biological Sciences Queen's University Belfast Belfast UK

**Keywords:** animal behaviour, environment framing, head movement, head pitch, head yaw, orientation sphere

## Abstract

1. Animal behavior is elicited, in part, in response to external conditions, but understanding how animals perceive the environment and make the decisions that bring about these behavioral responses is challenging.
2. Animal heads often move during specific behaviors and, additionally, typically have sensory systems (notably vision, smell, and hearing) sampling in defined arcs (normally to the front of their heads). As such, head‐mounted electronic sensors consisting of accelerometers and magnetometers, which can be used to determine the movement and directionality of animal heads (where head “movement” is defined here as changes in heading [azimuth] and/or pitch [elevation angle]), can potentially provide information both on behaviors in general and also clarify which parts of the environment the animals might be prioritizing (“environmental framing”).
3. We propose a new approach to visualize the data of such head‐mounted tags that combines the instantaneous outputs of head heading and pitch in a single intuitive spherical plot. This sphere has magnetic heading denoted by “longitude” position and head pitch by “latitude” on this “orientation sphere” (O‐sphere).
4. We construct the O‐sphere for the head rotations of a number of vertebrates with contrasting body shape and ecology (oryx, sheep, tortoises, and turtles), illustrating various behaviors, including foraging, walking, and environmental scanning. We also propose correcting head orientations for body orientations to highlight specific heading‐independent head rotation, and propose the derivation of O‐sphere‐metrics, such as angular speed across the sphere. This should help identify the functions of various head behaviors.
5. Visualizations of the O‐sphere provide an intuitive representation of animal behavior manifest *via* head orientation and rotation. This has ramifications for quantifying and understanding behaviors ranging from navigation through vigilance to feeding and, when used in tandem with body movement, should provide an important link between perception of the environment and response to it in free‐ranging animals.

## Graphical abstract

We provide a new visualization of animal body and head orientation that shows in an intuitive manner how the animal frames the environment. Metrics derived from this should help us understand how animals make decisions to adopt particular behaviours.

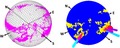

## 1 INTRODUCTION

Animal behavior may be viewed as a response to both internal state and environmental conditions, ultimately leading to enhanced lifetime reproductive success (cf. Nathan et al., 2008). Studies of behavior are many (Huntingford, 2012; Sih et al., 2015), and there are also extensive studies on how animals behave and move according to environmental circumstances (Lea, 2015; Snell‐Rood, 2013; Steinmeyer, Schielzeth, Mueller, & Kempenaers, 2010). However, the process by which information is perceived from the environment by an animal is less clear. Despite the rich and varied literature on animal sensory systems (Ali, 2013; Dusenbery, 1992; Fay & Tavolga, 2012; Martin, 2017), there is little research on exactly how free‐living animals orientate their sensory systems, located primarily on the head, toward areas in the environment of interest and, as a consequence, understanding the cues used by animals in navigation continues to be challenging (Doherty & Driscoll, 2018). However, the increasing use of head‐ and eye‐tracking technology in captivity using head‐mounted equipment (e.g., Williams, Mills, & Guo, 2011) means that developments in technology should now enable us to examine the head movements of wild animals. Indeed, despite reservations that such systems are likely to be suitable for wild animals moving uninhibited in their natural environment (Duchowski, 2017), Kano, Walker, Sasaki, and Biro (2018) have already demonstrated the use of a head‐tracking system on free‐flying homing pigeons, while Kane and Zamani (2014) used head‐mounted cameras to investigate how trained falcons hunt prey using visual motion cues. It therefore seems clear that similar systems will be used on wild animals soon (Kano, 2019). This will be made more likely by the increasing number of studies using head‐mounted technology of any type on wild animals, including head‐mounted accelerometers and magnetometers to quantify foraging success (e.g., Kokubun, Kim, Shin, Naito, & Takahashi, 2011; Watanabe & Takahashi, 2013; Ydesen et al., 2014). Attempts to quantify how animals might perceive their environment seems to have begun with human‐based approaches, most notably eye‐tracking technology (Duchowski, 2007; cf. Williams et al., 2011). The sophistication of such methods is, however, particularly challenging for wild animals, so simpler systems based on head, rather than eye, orientation (Wilson, Holton, et al., 2015; Wilson, Norman, et al., 2015) have proven useful on captive, but free‐flying, birds (Kane & Zamani, 2014; Kano et al., 2018). Indeed, these systems have revealed important details about how homing pigeons allocate their attention to the environment. Although this technology does not specifically allow researchers to pinpoint exactly where the eyes might be looking, because eyes can move relative to the head within their sockets, it does show how the subject “frames” the environment (Wilson, Norman, et al., 2015). The extent to which the environment is framed should encompass everything perceived by the visual system, although certain parts will receive more attention (due, e.g., to fovea). The orientation of the head thus determines which part of the environment is framed and therefore which part provides information from a visual perspective (Wilson, Norman, et al., 2015). The environmental framing approach has particular value for wild animals because many species use multiple, complex sensory systems with a weighting that does not necessarily reflect the high visual bias apparent in humans. In this regard, many vertebrates have sensory systems mounted on the head with a preponderance orientated frontally. However, this is less important that appreciating the arcs over which vertebrate sensory systems operate, which may equally be laterally (cf. Martin, 2017) and workers need to be cognisant of this in their interpretation of head orientation. As such though, determination of head orientation using the “environmental framing” concept should prove particularly valuable to the study of awareness in free‐ranging animals. Beyond this, because head movement is symptomatic of behaviors not involved with perception of the environment, such as grazing in ungulates (Ensing et al., 2014) or displays in birds (Tinbergen & Moynihan, 1952), quantification of head movement can help define behaviors that might otherwise be difficult to observe or even resolve using body‐mounted tags alone (Wilson et al., 2017).

A particular difficulty in the use of head orientation‐determining systems (HODS), however, is that the data that are collected represent the orientation of the head in more than one dimension. Specifically, the data are made up of head heading (or azimuth) in one plane and head pitch (also called elevation or dip angle) in another, perpendicular to this. Head roll (bank angle) is a third plane, although it generally changes gaze orientation minimally and is less likely to be more important than heading and pitch in environmental framing (but see Kano et al., 2018). We propose that these metrics can be conveniently examined in a single spherical visualization that we term the “orientation sphere” (O‐sphere) (cf. similar representations for visual field data (Martin, 2017)). Here, if we imagine that the spherical plot is represented by the Earth, the head heading is represented by the longitude while the head pitch is represented by the latitude. Thus, any head orientation is represented on the sphere by a single point that can be defined in terms of latitude and longitude (head pitch and head heading) and changes in head orientation will be represented by a trajectory across the sphere. In this single visualization, movement of the head can be inspected in a way that explicitly links both pitch and heading together better than as independent visualizations. This same visualization can also be used to show the orientations of animal bodies, which can be used as a stand‐alone method to aid in identification of behavior or to allow correction for the body so that head orientation can be examined relative to the body.

We present the methodology behind the building and interpretation of the O‐sphere and propose metrics that can be derived from these data to quantify the head movements in animals, thereby helping identify head‐focussed behaviors and quantify behaviors involved in environmental framing. As part of this, we present a modified methodology, developed from a recently published methodology, to identify turns in movement paths (Potts et al., 2018). We illustrate our concepts using data from head‐ and body‐mounted systems deployed on domestic sheep (*Ovis aries*), Arabian oryx (*Oryx leucoryx*), loggerhead turtles (*Caretta caretta*) undergoing rehabilitation, and wild Aldabra giant tortoises (*Aldabrachelys gigantean*).

## 2 MATERIALS AND METHODS

### 2.1 The head orientation‐determining system

The head orientation‐determining system (HODS) is based on the “Daily Diary” (DD) tag presented by Wilson, Shepard, and Liebsch (2008), consisting primarily of a logging unit linked to a tri‐axial (orthogonal axes) accelerometer and, similarly, tri‐axial magnetometer (although a more sophisticated version incorporating gyros for very fast‐moving animals (Johnson & Tyack, 2003) can also be used. These tags are typically used to record at frequencies of 10 Hz or greater. We assume that the tag is placed on the head so that the three acceleration axes represent the longitudinal (surge) axis, the dorso‐ventral (heave) axis, and the lateral (sway) axis of the head (see below). Depending on the axis/axes about which the tag is rotated, rotation of the tag (head) produces a change in the pitch (also called elevation or dip) angle and/or a change in the roll (also called lateral inclination) angle.

In the absence of roll, tag pitch angle (and by extraction, head pitch—see below) can be determined by taking a running mean of the longitudinal (surge) acceleration using a window of around 2 s (Shepard et al., 2008) and then taking the sine of the smoothed *g*‐values (1 g = 9.81 m.s^‐2^). Note that the relationship between the *g*‐value of acceleration and tag angle follows a sine wave. Therefore, the most accurate determinations of tag pitch angle for values around the horizontal (−45° to 45°) are derived by using the longitudinal acceleration axis (because this is when the rate of change of *g*‐value is greatest with respect to angular change). When the tag rolls however, calculation of its pitch is given by

$$\text{Pitch}=\text{atan}\left(\text{surge}/\left({\left({\text{heave}}^{2}+{\text{sway}}^{2}\right)}^{0.5}\right)\right)$$

where surge, heave, and sway are the acceleration values for those axes. There are packages in R, including those of animalTrack (Farrell, Fuiman, & Farrell, 2013), and software including DDMT (Wildbyte Technologies, http://wildbytetechnologies.com/software.html) that calculate both pitch and roll. Note that DDMT smooths the acceleration channels to determine pitch and roll parameters for levelling the magnetometer data (see below). This allows the algorithm to remove any transients that might be present and, at the same time, better represents more static values with the acceleration vector magnitude *sqrt(Acc_surge_^2^ + Acc_sway_^2^ + Acc_heave_^2^)* being closer to 1 g when measuring the device orientation compared to gravity. We note though, when animals are turning so rapidly that they are subject to substantial centripetal acceleration, that the acceleration vector magnitude may deviate appreciably from 1 g making calculations of pitch and roll invalid, with knock‐on consequences for derivation of heading (see below).

Tag heading is more complex. Its calculation uses all tri‐axial data from the magnetometers and the accelerometers within the HODS. The heading, pitch, and roll calculation is based on the well‐known “tilt‐compensated compass” algorithm. After correcting the magnetometry data for any hard/soft iron offsets and distortions, the accelerometer‐derived pitch axis and the roll axis are used to level the magnetometer data to the ground plane. Heading can then be calculated by using the magnetometer axes in the ground plane. Extensive detail on the process is provided by numerous authors including Bidder et al., (2015) and Koo, Sung, and Lee, (2009), and there is an R package (TrackReconstruction; Battaile, 2015) that undertakes this and a detailed code provided by Liu, Battaile, Trites, and Zidek (2015). The full process of disentangling heading from pitch and roll replies on spherical trigonometry and is detailed, for example, in Benhamou (2018).

### 2.2 Mounting the head orientation‐determining system

**Figure 1:**
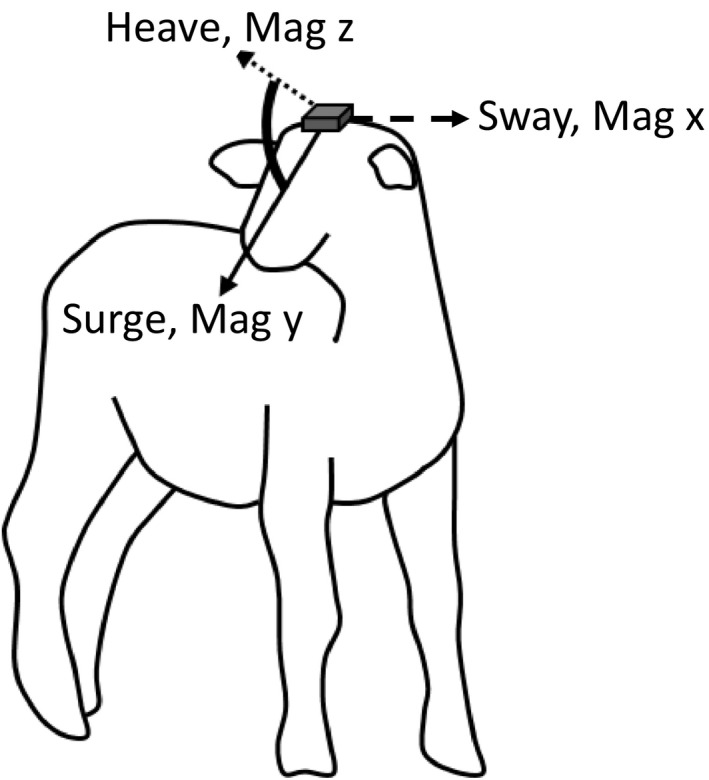
Placement of a head orientation‐determining system (HODS) on the head of an ungulate such as a sheep. The tag contains orthogonally placed tri‐axial accelerometers and magnetometers that should be aligned to represent the main axes of the head corresponding to the longitudinal (surge) axis, the dorso‐ventral (heave) axis, and the lateral (sway) axis

Ideally, the HODS should be set at the exact center of rotation of the mobile (head or body; see later) so that rotations do not produce translation. However, this is obviously impractical so workers should at least mount the unit on the head in a roughly central position, but so that the three orthogonal axes for both tri‐axial magnetometers and tri‐axial accelerometers align with the main orientation of the skull. Thus, one axis aligns with the longitudinal axis of the head, one with the dorso‐ventral axis of the head, and one with the lateral axis. For this, the tag can be placed on the top or the back of the head (e.g., Figure 1). In our work, we used glue to mount the HODS to the dorsal surface of the head of the oryx, sheep, and turtle and Velcro for the tortoise and used either glue or a collar to attach tags to animals' bodies (Supporting information S1). Glue only allows HODS to be retained until animal fur is molted and thus, in the event that tagged animals were not recaptured, would only maintain devices on animal heads for a maximum period of one year although this would normally not exceed a few weeks.

### 2.3 Construction of the O‐sphere

**Figure 2:**
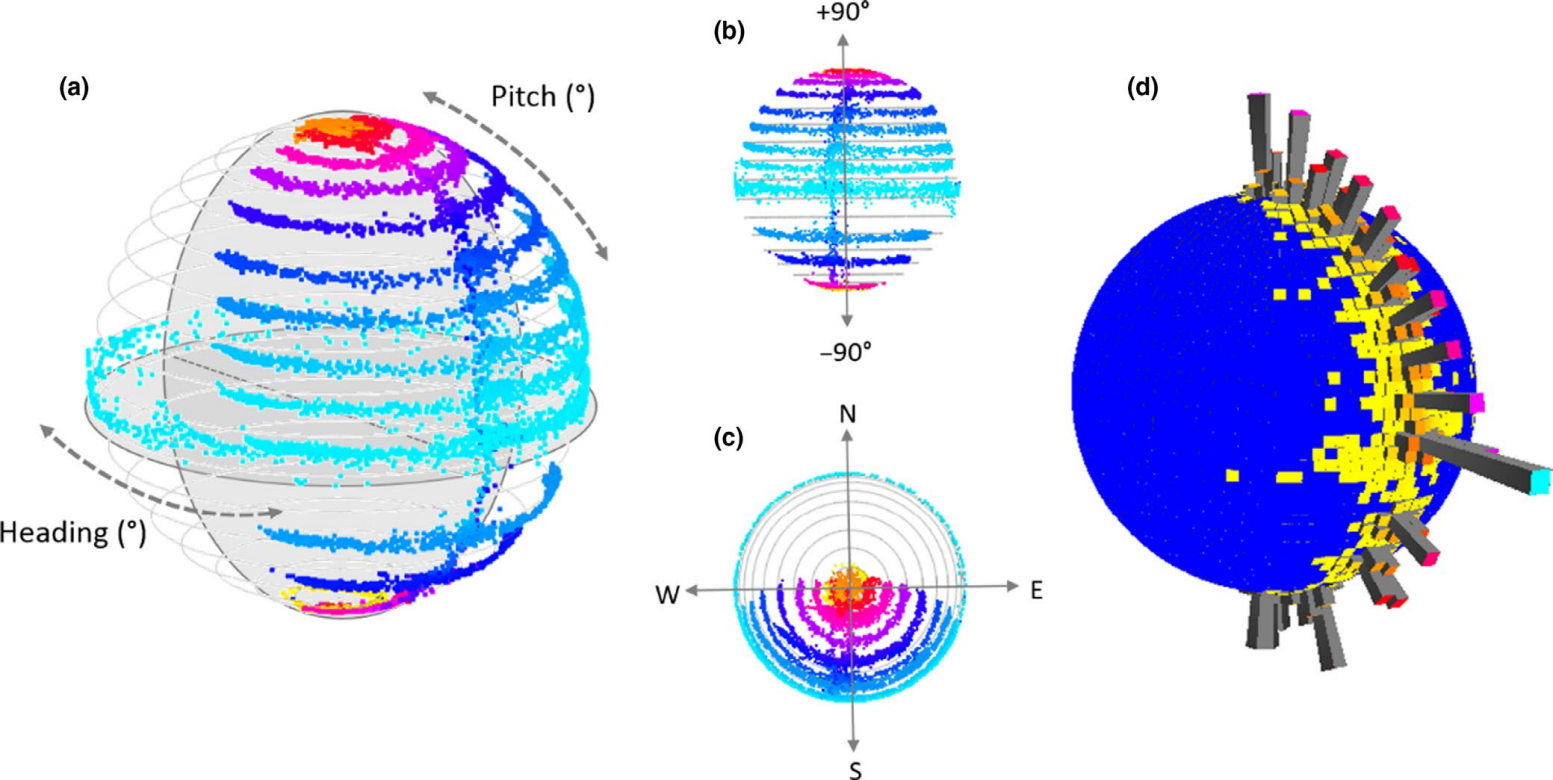
Representation of the orientation sphere (O‐sphere): When the HODS is placed on a level surface with zero pitch and rotated about its vertical axis, the O‐sphere consists of a ring of data at the equator (the lightest‐blue ring in (a)—points are colored by pitch with warmer colors increasingly deviating from zero degrees). Repeating this process at various angles of pitch in 10‐degree increments produces arcs at lower and higher latitudes (blue to red arcs, respectively, in (a, b) [highlighting pitch changes], and (c) [highlighting heading changes]). (d) These same data can also be represented on the O‐sphere as a histogram (a 'Dubai plot'), which depicts the time‐based O‐sphere usage. In this representation, colors have been added to the ends of the bars to help differentiate the height and enhance an understanding of the 3D visualization

The heading data are represented by a circular plot spanning 0–360° and this is enhanced by a further circular plot running perpendicularly to show the head pitch (in degrees) corresponding to each head heading (Supporting information S2) (Figure 2). Since animals rarely have head pitches that are less than −90° (where e.g., the head looks back along and underneath the body) and exceed 90° (where the head is inverted to look along the top of the body), the O‐sphere plot is an intuitive 3‐dimensional plot that highlights head orientation with respect to heading and pitch.

In addition, because the data on the O‐sphere can be subject to occlusion and over‐plotting, extended time periods can be represented on the O‐sphere using a “Dubai” plot (Wilson et al., 2016). This shows point density over the surface of the sphere in the form of variable radiating height bars, where length is proportional to point density (Figure 2d). To do this, the surface of the O‐sphere is tessellated into facets, within which the number of data points is summed to inform the bar height metrics (for details on this process see Wilson et al., 2016).

### 2.4 Identifying turning points in head orientation

Within environmental framing, the orientation of the head directs sensory systems to derive input from a particular part of the environment and two head behaviors have been recognized as important in this for humans; “scanning,” and “object fixation” (Wilson, Norman, et al., 2015). Scanning involves head rotation in the pitch and/or yaw axes while fixation results in no head movement for a defined period (Blascheck et al., 2014), during which information is gained from an object or area of interest: The points at which scanning changes direction or becomes a fixation can be considered to be turning points on the O‐sphere.

In order to identify head‐based scanning and turning points (rather than overall [head and body] movements), both body and head orientation should be derived using compass tilt‐compensated systems (based on mathematical expressions relating yaw and pitch to the orthodromic arc size (Benhamou, 2018)). We note, though, that most terrestrial animals on the ground have a bank angle close to 0° together with a restricted pitch angle. To identify such turning points of the head, independent of the overall body movement, we first identify turning points in the head and the body separately, each in 3‐dimensional orientation space defined by head pitch and heading. Recent work has quantified turning angles in the heading plane to define decision points in the movement path. The algorithm for turning point identification is derived from the algorithm for inferring 2D turning points from Potts et al., (2018), but modified for 3D data. We slide a window of size W across a bivariate time series consisting of both heading and pitch. At each point i in time series, the Squared Spherical Standard Deviation (SSSD) across the sliding window is calculated. The SSSD is a version of variance that accounts for the spherical geometry and generalizes the squared circular standard deviation (e.g., Berens, 2009). More precisely, for any bivariate time series $\left\{\left(h_{1},p_{1}\right),\ldots ,\left(h_{N},p_{N}\right)\right\}$ where $h_{1},\ldots ,h_{N}$ are the headings and $p_{1},\ldots ,p_{N}$ are the pitch angles, the SSSD across the sliding window is given by

$$s_{i}=\ln\left(\frac{1}{R_{i}^{2}}\right)$$

where $R_{i}=\sqrt{<x_{i}{>}^{2}+<y_{i}{>}^{2}+<z_{i}{>}^{2}}$ , $x_{i}=\cos\left(p_{i}\right)\cos\left(h_{i}\right)$ , $y_{i}=\cos\left(p_{i}\right)\sin\left(h_{i}\right)$ , $z_{i}=\sin\left(p_{i}\right)$ , and <*x_i_*>, <*y_i_*>, <*z_i_*> are, respectively, the means of the sets $\left\{x_{i-\frac{W}{2}},\ldots ,x_{i+\frac{W}{2}}\right\}$ , $\left\{y_{i-\frac{W}{2}},\ldots ,y_{i+\frac{W}{2}}\right\}$ and $\left\{z_{i-\frac{W}{2}},\ldots ,z_{i+\frac{W}{2}}\right\}$ (per force, W must be an even integer).

Following Potts et al., (2018), we define a *spike* in the SSSD of a bivariate time series to be a contiguous set of points where the SSSD is above the global mean. The midpoint of a spike is defined to be a candidate turning point. This set of candidate turning points is then refined further by removing any points where the turn is less than a threshold angle $\theta_{\mathrm{thresh}}$ , in an identical fashion to the method of Potts et al., (2018).

To illustrate the method, we identified turning points in the orientation of the body and head of the sheep from a collar and HODS, respectively. These turning points were defined over a 40 event (corresponding to 1 s since the tags were recording at 40 Hz) window and at a threshold of 30 degrees turn.

## 3 RESULTS

### 3.1 Head orientation with a static animal body

**Figure 3:**
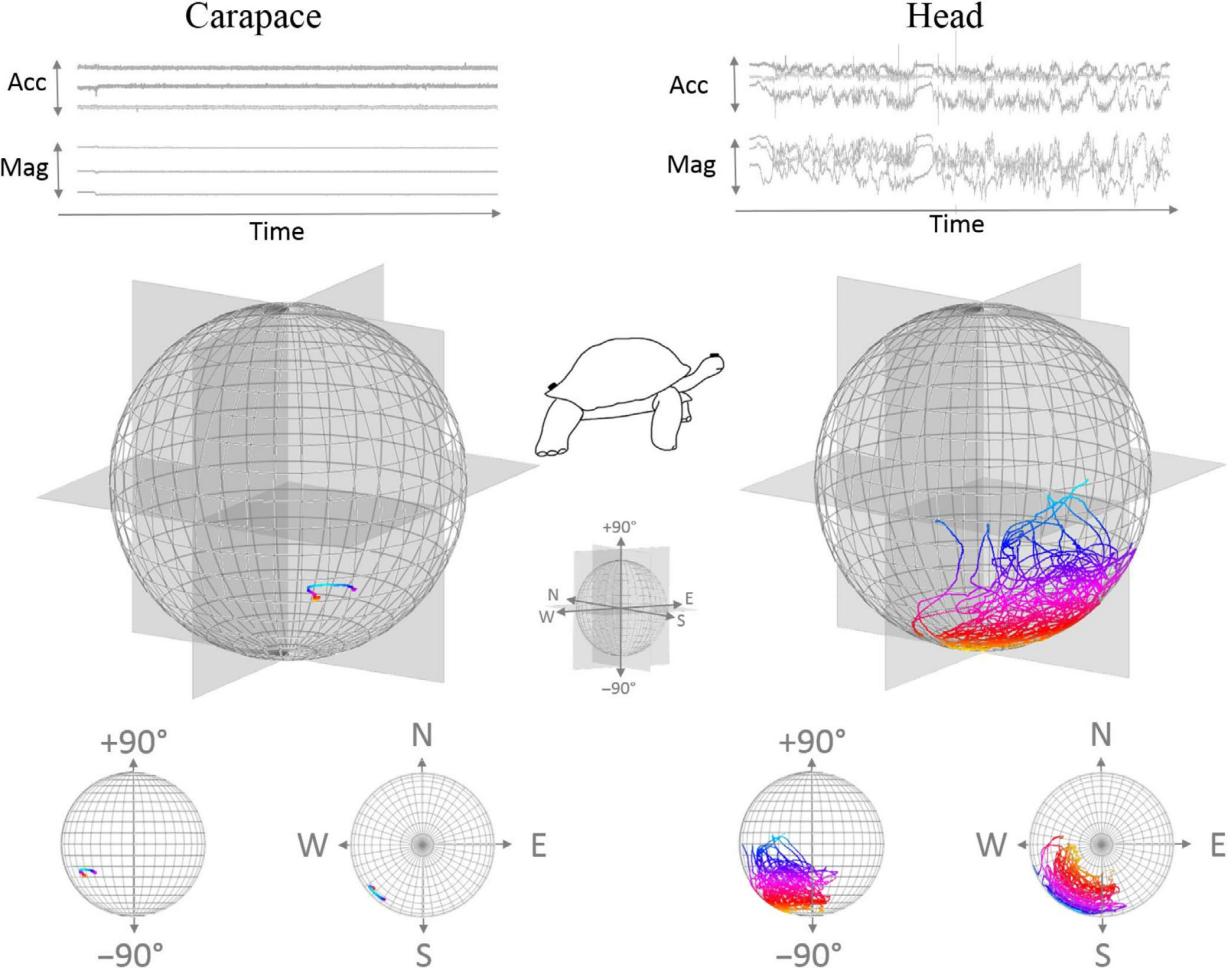
Body and head orientation of an Aldabra tortoise over 20 min derived using carapace‐ and head‐mounted tags (corrected for a “pitch‐up” angled carapace‐mounted tag to read 0° when the animal was level but showing slight “pitch‐down” attitude due to the animal feeding on an incline). (a) shows a time series of tri‐axial accelerometer (Acc) and magnetometer (Mag) data while (b) shows the same data resolved into pitch and yaw and plotted onto O‐spheres. (c) shows planar plots of the O‐sphere to allocate pitch and heading more easily as separate entities. To aid visualization, points are colored by pitch, with warmer colors increasingly deviating from the horizontal at 0°. Note that the body moves little while the head covers a pitch range in excess of 90°, predominantly directed down, and a heading difference of about 160°

The O‐sphere visualizations provided an intuitive representation of head orientation, which is especially apparent when the animal is static but the head active. For example, in the case of the foraging Aldabra tortoise, time series plots of the carapace‐attached tag (recording at 40 Hz) show extremely little change in body posture or orientation and yet over the same time period, a head‐mounted tag (sampling at 40 Hz) showed extensive rotation in pitch and heading (Figure 3). When visualized on the O‐sphere, this variation in head orientation becomes clear. Specifically, the head tended to operate between level and negative pitch while the head heading moved over some 160*°*. The movements of the head are due to the animal extending its neck left and right, and down and up, to exploit the vegetation within reach with minimal movement of the body. This demonstrates how the flexibility of the neck of this species, allows it to gain energy without paying the extensive power costs associated with moving the body (cf. Wilson et al., 2017).

### 3.2 Head orientation with a dynamic animal body

**Figure 4:**
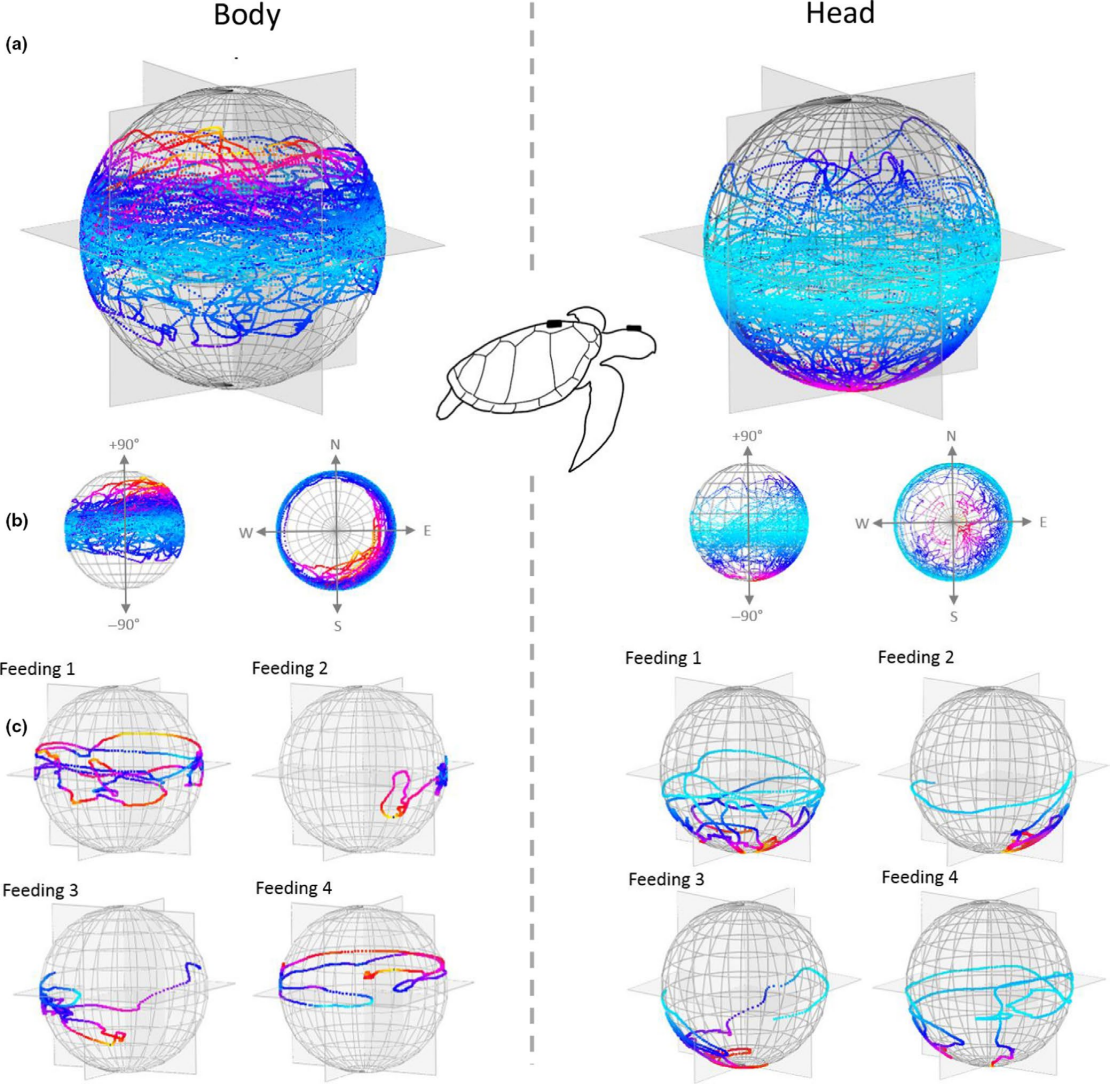
O‐sphere visualizations of a foraging loggerhead turtle over 20 min during a series of feeding experiments in circular tanks (ranging from 2 to 6 m in diameter with a water depth of 0.95 m). (a) shows all data for both the body (tag attached to the carapace) and the head with (b) showing planer views of the same data to highlight pitch and heading variation. (c) shows the detail for each of four events where the animal was consuming green crabs (Carcinus maenas) located at random locations and presented at 5‐min intervals. To aid visualization, line is colored by pitch, with warmer colors increasingly deviating from the horizontal at 0°. Note how body pitch is constrained compared to the head that was particularly pitched down to access the crab on the tank bottom

Movement of the head is not the only mechanism by which an animal can orientate its sensory system to frame the environment because the body also plays a role. In our example animals, the O‐spheres of the heads of moving animals typically had greater variability than those of stationary animals, and this is shown by comparison of feeding between the Aldabra tortoise and the loggerhead turtle (Figures 3 and 4). Both species had highly active heads, but the dynamism of the turtle head O‐sphere was partially due to the movement of the body as well as that of the head, with the ultimate head orientation effectively depending on the body orientation and the head orientation with respect to the body. Accordingly, in the turtle example, the orientation of the head clearly showed greater range of pitch than the body (Figure 4).

### 3.3 Turn points in the head movement

**Figure 5:**
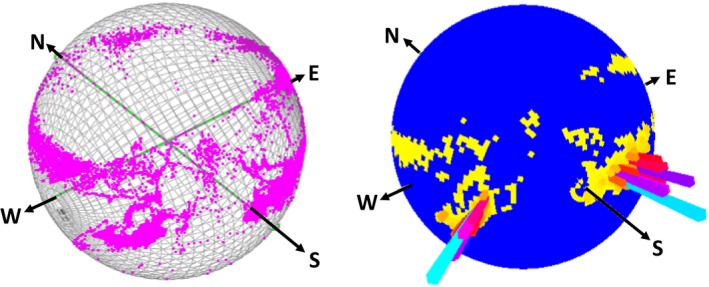
Two O‐spheres depicting the same ca. 3 min of Arabian oryx head behavior. The left‐hand sphere clearly shows that the animal rotated through 360° (circles of points running between N‐W‐E in the diagram) but that more time was spent facing particular headings. The right‐hand sphere shows the same data in a “Dubai” plot, which resolves superimposed data into histograms that make clear the orientations where the head position was fixed for extended periods

**Figure 6:**
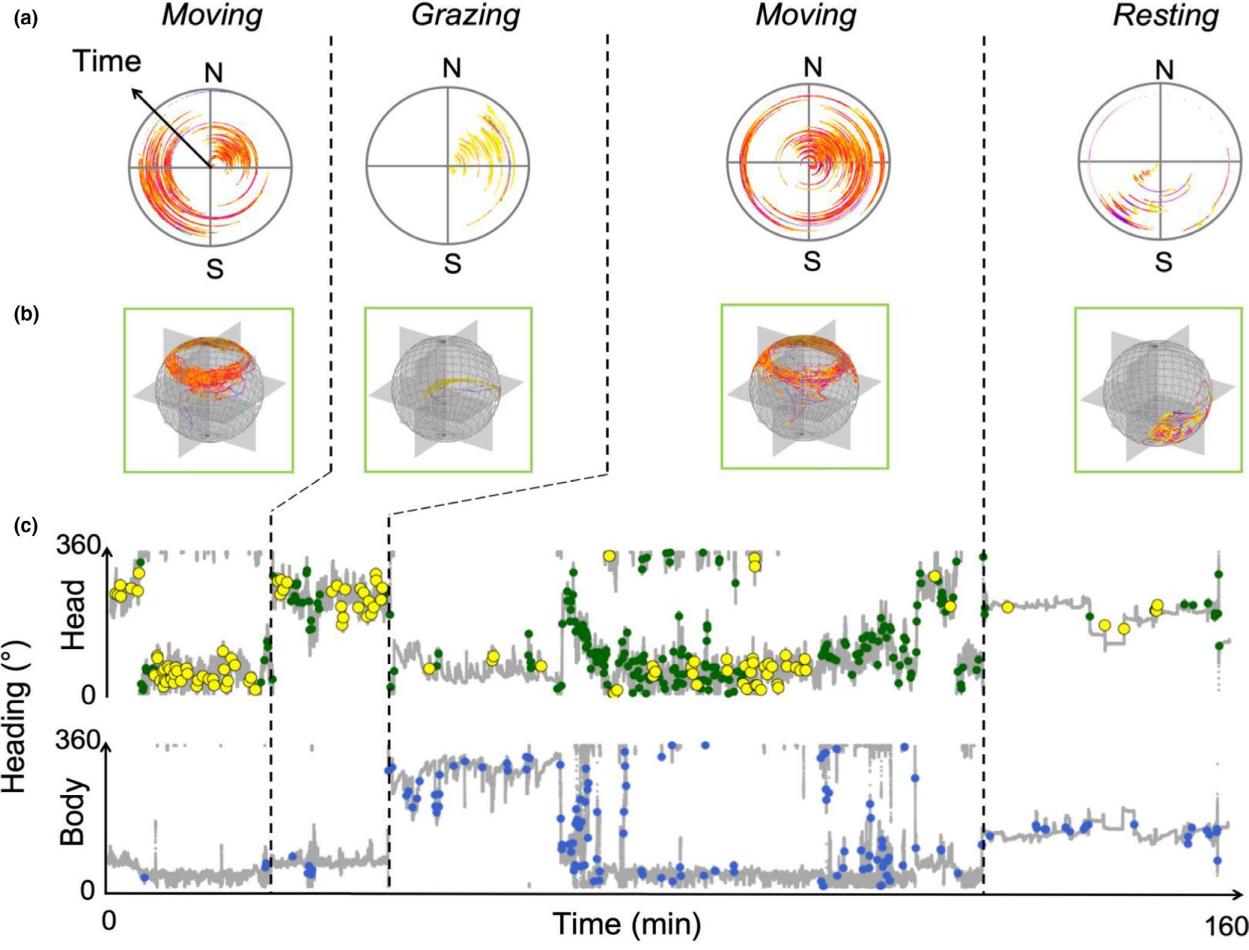
Turning points on the O‐sphere identified using the SSSD‐spike method (see text) applied to a 160‐min dataset of a sheep fitted with head and body tags performing different behaviors (moving, resting and grazing). Row (a) shows a circular plot for head heading with time development represented by increasing radius (with yellow showing head pitched down, through orange and red to purple showing head INCREASINGLY pitched up), (b) shows the same data on O‐spheres while (c) shows the head and body headings over time in a conventional plot. In (c), the turn points in the head (green circles) generally concur with the turn points in the body (blue circles) except for those points in yellow which highlight turn points of the head that are not mirrored by the body. Note how few yellow points occur when the animal is resting compared to moving (when the sheep scans the environment and fixates on relevant parts as it travels)

We noted that head O‐spheres manifest three primary “behaviors.” The first of these was “fixations” (Blascheck et al., 2014), these fixations being particularly apparent in Dubai plots of the O‐sphere (Figure 5). Secondly, especially when fixations were short, heads showed “transitions” between fixations, where the head moved from one point on the O‐sphere to another (e.g. angular velocities were around medians of *ca*. 4.6°/s (interquartile range (IQR) 6.1) for feeding tortoises and 4.6°/s (IQR 11.9) for feeding turtles). Finally, “scans” occurred, where the head moved continuously, and relatively slowly (e.g., median angular velocities were *ca*. 5.2°/s (IQR 7.1) for walking sheep) across the sphere. These movements typically occurred either horizontally or vertically and could, for example, oscillate from one side to the other. The change‐point approach described above readily highlighted patterns of head movement, both for animal activity in general (Figure 6, cf. Figure 5) and for specific stylized movements such as head movement during walking (see later).

**Figure 7:**
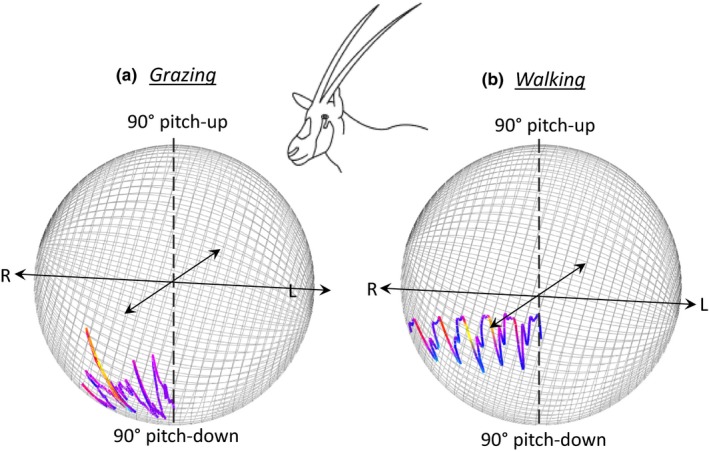
Body relative, time‐based O‐spheres showing how the visualization highlights key features during 5 s of (a) grazing and (b) walking by an Arabian oryx. The spheres show the head angles with respect to the body (head pitch and yaw)—which takes true heading out of the O‐sphere. The spheres also show the progression of time by having time start at a sphere radius of 0, expanding out linearly (cf. Figure 6a). Finally, the angular speed in the yaw axis is color‐coded with colder colors showing slower speeds (each sphere color codes within maximum limits for the period shown). Note how each behavior is exemplified by a stylized trajectory across the O‐sphere with a consistent pattern in the angular speed. This 4‐dimensional representation is best visualized if the image can rotate (see Supplementary Video S1)

The interplay of the body‐ with respect to the head directionality in O‐sphere plots is useful in cases where heading is specifically relevant (grazing being one—Figure 3) but superfluous and liable to hide patterns in others. This is because variation in animal orientation with respect to North may superimpose patterns indicative of behavior as the animal rotates. To remove this influence, where recorded, the body heading (derived using the tilt‐compensated compass) can be simply subtracted from the head heading to give head yaw on the “body relative O‐sphere.” This negates animal body directionality and makes particular patterns from stylized behaviors, such as walking, more apparent (Figure 7).

## 4 DISCUSSION

Head pitch and heading (including changes in these two parameters) highlights many behaviors clearly and we suggest that the O‐sphere helps in visualization and interpretation, most particularly because it integrates heading/yaw with pitch. For the convenience of this work, behaviors may be grouped into; (i) “general behaviors,” which range from extended static positions during e.g. sleep (with O‐sphere Dubai plots manifesting single, high bar histograms; cf. Figure 5) to repetitive movement across the O‐sphere e.g. as occurs in feeding (apparent in our tortoise study, Figure 3e) or during stylized displays (e.g., Hester, Gordon, Baillie, & Tappin, 1999; Périquet et al., 2010), which may be particularly conventionalized in birds (Frith, 1992) and (ii) behavior associated with environmental framing, manifesting scanning and fixations (cf. Figure 5). As with stylized displays, such environmental framing is liable to be short‐term, resulting in transitions across the O‐sphere interspaced with fixations (Figure 5) (Wilson, Norman, et al., 2015). These can be seen and inspected on the O‐sphere but, most importantly, the O‐sphere specifically provides a platform from which metrics of particular behaviors can be defined. These include, but are not limited to, rates of change of pitch/heading across the O‐sphere (cf. Figure 7), the rates of change of pitch/heading with respect to co‐ordinates on the O‐sphere (Figure 7), probabilistic occupation of certain co‐ordinates on the O‐sphere (Figures 5 and 7), the temporal sequence of trajectories across the O‐sphere (Figure 7), the co‐ordinates of turn points (Figure 6), and the length of stationary periods on the O‐sphere (Figure 5). Such an approach provides important discriminatory metrics to machine‐learning methods (e.g., Random Forests (Breiman, 2001)), but also to Boolean methods for identifying behaviors (Wilson et al., 2018) and should increase the number of behaviors that can be reliably isolated using data from animal‐attached tags. Of particular value is the ability to define stylized head movements precisely. These include for example, feeding (Hester et al., 1999), vigilance (Périquet et al., 2010), acquisition of social information (Colagross & Cockburn, 1993) and navigation (Caduff & Timpf, 2008). At the same time, examination of the O‐sphere should allow workers to have more intuitive contact with the data than might occur with machine‐learning alone.

### 4.1 Varying the length of time of the O‐sphere representation

The value of the O‐sphere as a visualization tool depends critically on the length of time covered in any particular visualization. Shorter periods clearly show transient head behavior well, but this becomes less apparent with increasing time intervals. The specific lengths of time to achieve particular objectives will also depend on the study animal (tortoises obviously move their heads slower than mammals). Since the length of fixations on the O‐sphere is likely to indicate whether animals are inspecting sites of interest within the environment (cf. Wilson, Norman, et al., 2015), resting or for example, ruminating (something that is considered particularly problematic to identify using tag technology (di Virgilio, Morales, Lambertucci, Shepard, & Wilson, 2018)), we suggest that workers separate “active” from “inactive” periods (Watanabe, Izawa, Kato, Ropert‐Coudert, & Naito, 2005). This can be done effectively if an additional body‐mounted tag records acceleration (e.g., Wilson et al., 2008) so that for example, dynamic body acceleration (e.g., Qasem et al., 2012) can code for activity (Brown, Kays, Wikelski, Wilson, & Klimley, 2013).

### 4.2 Body versus head movement for the O‐sphere

Any activity that changes the orientation of the body will tend to change that of the head although the head is usually stabilized to an appreciable degree (Pozzo, Berthoz, & Lefort, 1990). Importantly though, simple examination of head orientation using the O‐sphere will tend to have greater lability and translation of points across the O‐sphere due to the signal being a composite of the head and the body (cf. Figures 3 and 4—but see stabilization of the head in birds (Frost, 2009) and Kano et al., 2018). In addition, moving animals normally have particular reason to inspect the environment more thoroughly than stationary ones (which may mean more short‐term fixations and higher transition rates). This behavior helps them move safely through the environment, both with respect to the biomechanical issues of moving over the terrain and the possibility of predation in an environment that is continuously revealing itself as the animal moves. Interpretation of the patterns on the O‐sphere need to bear this in mind, and workers may consider correcting head‐based heading (and pitch) with respect to that of the body (which could also be represented by a relative O‐sphere) to compare head movement with that of the body. Importantly though, even if the O‐sphere is considered without reference to the body orientation, the data should still indicate which parts of the environment are potentially being examined by the animal (as evident in the tortoise and turtle O‐spheres; Figures 3 and 4).

### 4.3 The O‐sphere and environmental context

Perhaps the most powerful use of the O‐sphere becomes apparent when it is placed into environmental context. In a general sense, feeding behavior in some species relates to the head dealing with food lower than the body (Figures 3 and 4), but modern animal‐attached technology can use GPS systems or GPS‐enabled dead‐reckoning systems to give remarkable detail on an animal's position in geo‐referenced space (Bidder et al., 2015; Tomkiewicz, Fuller, Kie, & Bates, 2010). At a coarse level, this approach can provide O‐sphere metrics that can then be allocated to environment types (cf. Figure 6), but at a more refined level it may be possible to determine which features of the landscape are being inspected. For example, we might expect animals to be more vigilant ‐ displaying specific head orientation directed toward certain areas coupled with characteristic movement behavior—in areas where predators can hide (di Virgilio et al., 2018). Similarly, other behaviors which may be identified using an O‐sphere approach, such as feeding (Figures 2, 3, and 6) (di Virgilio et al., 2018), grooming (Mooring, Blumstein, & Stoner, 2014) and highly specialized behaviors, such as head shaking in primates (Schneider, Call, & Liebal, 2010), could be allocated to the specific geographical areas where the behavior was displayed.

There are undoubted difficulties with interpretation of HODS data, and the O‐sphere is just one convenient and promising approach. However, the potential in such systems for informing us about the features of the environment that might be affecting animal behavior makes the subject area exciting. A particular example might be allocating fixations to specific points within the environment and then determining how the finely resolved animal track (e.g., Bidder et al., 2015), or other behavior, has changed (or not), as a result of the information gathered during such fixations. Indeed, the ability to trace the specific element of the environment that has driven the manifestation of a particular behavior is the next exciting step in linking behavior and energy expenditure to circumstance.

## 5 CONCLUSIONS

Head‐mounted tags from which head pitch and heading can be deduced, produce much complex information which codes for the way the animal frames the environment and behaves. The O‐sphere is an intuitive plot that distils out, and links, head heading and pitch in one 3D plot, which can be extended to 4 dimensions if time is introduced as a radial axis, and 5 dimensions if the trajectory across the O‐sphere is color‐coded for angular speed. This enables researchers to have, at once, an impression of changes in behavior while providing metrics that can be used in conventional methods for identifying them. The specific strength of O‐spheres in highlighting environmental framing behaviors as well as other behavior types means that we may be nearing a time when we can specifically link environmental context to behavioral response.

## AUTHORS' CONTRIBUTIONS

HJW, MH and RPW: Conceptualizationand development of the methodology, with input from all other authors. AA, AdV, AbA, DMS, NCK and LB: Collection of the data. AA, AdV, HJW, JRP, LB, and RG: Analysis of the data. All authors contributed critically to the development of, and writing, the manuscript.

## Supporting information

Supplementary Material

Video S1

## Data Availability

All data will be archived in Figshare under DOI; 10.6084/m9.figshare.11903178.

## References

[ece36197-bib-0001] Ali, M. (2013). Sensory ecology: review and perspectives. In Ali M. (Ed.), New York, NY: Springer Science & Business Media.

[ece36197-bib-0002] Battaile, B. (2015). R Package ' TrackReconstruction '. Retrieved from https://cran.r-project.org/package=TrackReconstruction

[ece36197-bib-0003] Benhamou, S. (2018). Mean squared displacement and sinuosity of three‐dimensional random search movements, arXiv, 1801.02435. https://arxiv.org/abs/1801.02435

[ece36197-bib-0004] Berens, P. (2009). CircStat: A MATLAB toolbox for circular statistics. Journal of Statistical Software, 31, 1–21. 10.18637/jss.v031.i10

[ece36197-bib-0005] Bidder, O. R. , Walker, J. S. , Jones, M. W. , Holton, M. D. , Urge, P. , Scantlebury, D. M. , … Wilson, R. P. (2015). Step by step: Reconstruction of terrestrial animal movement paths by dead‐reckoning. Movement Ecology, 3(1), 1–16. 10.1186/s40462-015-0055-4 26380711 PMC4572461

[ece36197-bib-0006] Blascheck, T. , Kurzhals, K. , Raschke, M. , Burch, M. , Weiskopf, D. , & Ertl, T. (2014). State‐of‐the‐art of visualization for eye tracking data. Proceedings of Eurographics Conference on Visualisation In: Borgo R., Maciejewski R., & Viola I. (Eds.), State of the Art Report. Norrköping, Sweden: The Eurographics Association.

[ece36197-bib-0007] Breiman, L. (2001). Random forests. Machine Learning, 45(1), 5–32.

[ece36197-bib-0008] Brown, D. D. , Kays, R. , Wikelski, M. , Wilson, R. , & Klimley, A. P. (2013). Observing the unwatchable through acceleration logging of animal behavior. Animal Biotelemetry, 1, 1–16. 10.1186/2050-3385-1-20

[ece36197-bib-0009] Caduff, D. , & Timpf, S. (2008). On the assessment of landmark salience for human navigation. Cognitive Processing, 9(4), 249–267. 10.1007/s10339-007-0199-2 17999102

[ece36197-bib-0010] Colagross, A. M. L. , & Cockburn, A. (1993). Vigilance and grouping in the eastern gray kangaroo, Macropus Giganteus. Australian Journal of Zoology, 41(4), 325–334. 10.1071/ZO9930325

[ece36197-bib-0011] di Virgilio, A. , Morales, J. M. , Lambertucci, S. A. , Shepard, E. L. C. , & Wilson, R. P. (2018). Multi‐dimensional Precision Livestock Farming: a potential toolbox for sustainable rangeland management. PeerJ, 6, e4867 10.7717/peerj.4867 29868276 PMC5984589

[ece36197-bib-0012] Doherty, T. S. , & Driscoll, D. A. (2018). Coupling movement and landscape ecology for animal conservation in production landscapes. Proceedings of the Royal Society B: Biological Sciences, 285(1870), 20172272 10.1098/rspb.2017.2272 PMC5784197 29298935

[ece36197-bib-0013] Duchowski, A. T. (2007). Eye Tracking Methodology. Theory and Practice, 328(614), 2–3.

[ece36197-bib-0014] Duchowski, A. T. (2017). Diversity and Types of Eye Tracking Applications In Eye Tracking Methodology (pp. 247–248). Cham: Springer.

[ece36197-bib-0015] Dusenbery, D. B. (1992). How Organisms Acquire and Respond to Information. Sensory Ecology. New York, NY: WH Freeman and Co, New York.

[ece36197-bib-0016] Ensing, E. P. , Ciuti, S. , de Wijs, F. A. L. M. , Lentferink, D. H. , ten Hoedt, A. , Boyce, M. S. , & Hut, R. A. (2014). GPS based daily activity patterns in European red deer and North American elk (Cervus elaphus): Indication for a weak circadian clock in ungulates. PLoS ONE, 9(9), e106997 10.1371/journal.pone.0106997 25208246 PMC4160215

[ece36197-bib-0017] Farrell, E. , Fuiman, L. , & Farrell, M. E. (2013). Package 'animalTrack'.

[ece36197-bib-0018] Fay, R. R. , & Tavolga, W. N. (2012). Sensory biology of aquatic animals. New York, NY: Springer Science & Business Media.

[ece36197-bib-0019] Frith, C. B. (1992). Standardwing Bird of paradise *Semioptera* *wallacii* displays and relationships, with comparative observations on displays of other Paradisaeidae. Emu, 92, 79–86.

[ece36197-bib-0020] Frost, B. J. (2009). Bird head stabilization. Current Biology, 19(8), R315–R316. 10.1016/j.cub.2009.02.002 19409275

[ece36197-bib-0021] Hester, A. J. , Gordon, I. J. , Baillie, G. J. , & Tappin, E. (1999). Foraging behaviour of sheep and red deer within natural heather grass mosaics. Journal of Applied Ecology, 36(1), 133–146. 10.1046/j.1365-2664.1999.00387.x

[ece36197-bib-0022] Huntingford, F. (2012). The study of animal behaviour. New York, NY: Springer Science & Business Media.

[ece36197-bib-0023] Johnson, M. P. , & Tyack, P. L. (2003). A digital acoustic recording tag for measuring the response of wild marine mammals to sound. IEEE Journal of Oceanic Engineering, 28(1), 3–12. 10.1109/JOE.2002.808212

[ece36197-bib-0024] Kane, S. A. , & Zamani, M. (2014). Falcons pursue prey using visual motion cues: New perspectives from animal borne cameras. The Journal of Experimental Biology, 217, 225–234. 10.1242/jeb.092403 24431144 PMC3898623

[ece36197-bib-0025] Kano, F. (2019). What are flying birds looking at? New challenges in the use of cutting‐edge sensor technologies to study bird gaze. Japanese Journal of Animal Psychology, 61–69. 10.2502/janip.69.1.1

[ece36197-bib-0026] Kano, F. , Walker, J. , Sasaki, T. , & Biro, D. (2018). Head‐mounted sensors reveal visual attention of free‐flying homing pigeons. Journal of Experimental Biology, 221(17), jeb183475 10.1242/jeb.183475 30190414

[ece36197-bib-0027] Kokubun, N. , Kim, J.‐H. , Shin, H.‐C. , Naito, Y. , & Takahashi, A. (2011). Penguin head movement detected using small accelerometers: A proxy of prey encounter rate. Journal of Experimental Biology, 214, 3760–3767. 10.1242/jeb.058263 22031740

[ece36197-bib-0028] Koo, W. , Sung, S. , & Lee, Y. J. (2009). Development of real‐time heading estimation algorithm using magnetometer/IMU. ICCAS‐SICE IEEE, 2009, 4212–4216.

[ece36197-bib-0029] Lea, S. (2015). Instinct, environment and behaviour. London: Psychology Press.

[ece36197-bib-0030] Liu, Y. , Battaile, B. C. , Trites, A. W. , & Zidek, J. V. (2015). Bias correction and uncertainty characterization of Dead ‐ Reckoned paths of marine mammals. Animal Biotelemetry, 1–11, 10.1186/s40317-015-0080-5

[ece36197-bib-0031] Martin, G. (2017). The sensory ecology of birds. Oxford: Oxford University Press.

[ece36197-bib-0032] Mooring, M. S. , Blumstein, D. T. , & Stoner, C. J. (2014). The evolution of parasite‐defence grooming in ungulates. Biological Journal of the Linnean Society, 81, 17–37.

[ece36197-bib-0033] Nathan, R. , Getz, W. M. , Revilla, E. , Holyoak, M. , Kadmon, R. , Saltz, D. , & Smouse, P. E. (2008). A movement ecology paradigm for unifying organismal movement research. Proceedings of the National Academy of Science, 105(49), 19052–19059. 10.1073/pnas.0800375105 PMC2614714 19060196

[ece36197-bib-0034] Périquet, S. , Valeix, M. , Loveridge, A. J. , Madzikanda, H. , Macdonald, D. W. , & Fritz, H. (2010). Individual vigilance of African herbivores while drinking: The role of immediate predation risk and context. Animal Behaviour, 79(3), 665–671. 10.1016/j.anbehav.2009.12.016

[ece36197-bib-0035] Potts, J. R. , Börger, L. , Scantlebury, D. M. , Bennett, N. C. , Alagaili, A. , & Wilson, R. P. (2018). Finding turning‐points in ultra‐high‐resolution animal movement data. Methods in Ecology and Evolution, 9(10), 2091–2101. 10.1111/2041-210X.13056

[ece36197-bib-0036] Pozzo, T. , Berthoz, A. , & Lefort, L. (1990). Head stabilization during various locomotor tasks in humans. Experimental Brain Research, 82, 97–106. 10.1007/BF00230842 2257917

[ece36197-bib-0037] Qasem, L. , Cardew, A. , Wilson, A. , Griffiths, I. , Halsey, L. G. , Shepard, E. L. C. , … Wilson, R. P. (2012). Tri‐axial acceleration as a proxy for energy expenditure; should we be summing values or calculating the vector? PLoS ONE, 7(2), e31187. 22363576 10.1371/journal.pone.0031187 PMC3281952

[ece36197-bib-0038] Schneider, C. , Call, J. , & Liebal, K. (2010). Do bonobos say NO by shaking their head? Primates, 51, 199–202. 10.1007/s10329-010-0198-2 20419332

[ece36197-bib-0039] Shepard, E. , Wilson, R. P. , Halsey, L. G. , Quintana, F. , Gómez Laich, A. , Gleiss, A. C. , … Norman, B. (2008). Derivation of body motion via appropriate smoothing of acceleration data. Aquatic Biology, 4(3), 235–241. 10.3354/ab00104

[ece36197-bib-0040] Sih, A. , Mathot, K. J. , Moirón, M. , Montiglio, P.‐O. , Wolf, M. , & Dingemanse, N. J. (2015). Animal personality and state–behaviour feedbacks: A review and guide for empiricists. Trends in Ecology and Evolution, 30, 50–60. 10.1016/j.tree.2014.11.004 25498413

[ece36197-bib-0041] Snell‐Rood, E. C. (2013). An overview of the evolutionary causes and consequences of behavioural plasticity. Animal Behaviour, 85, 1004–1011. 10.1016/j.anbehav.2012.12.031

[ece36197-bib-0042] Steinmeyer, C. , Schielzeth, H. , Mueller, J. C. , & Kempenaers, B. (2010). Variation in sleep behaviour in free‐living blue tits, Cyanistes caeruleus: Effects of sex, age and environment. Animal Behaviour, 80, 853–864. 10.1016/j.anbehav.2010.08.005

[ece36197-bib-0043] Tinbergen, N. , & Moynihan, M. (1952). Head flagging in the Black‐headed Gull; its function and origin. Brit. Birds, 45, 19–22.

[ece36197-bib-0044] Tomkiewicz, S. M. , Fuller, M. R. , Kie, J. G. , & Bates, K. K. (2010). Global positioning system and associated technologies in animal behaviour and ecological research. Philosophical Transactions of the Royal Society of London B: Biological Sciences, 365, 2163–2176. 10.1098/rstb.2010.0090 20566494 PMC2894966

[ece36197-bib-0045] Watanabe, S. , Izawa, M. , Kato, A. , Ropert‐Coudert, Y. , & Naito, Y. (2005). A new technique for monitoring the detailed behaviour of terrestrial animals: A case study with the domestic cat. Applied Animal Behaviour Science, 94, 117–131. 10.1016/j.applanim.2005.01.010

[ece36197-bib-0046] Watanabe, Y. Y. , & Takahashi, A. (2013). ). Linking animal‐borne video to accelerometers reveals prey capture variability. Proceedings of the National Academy of Sciences of the United States of America, 110(6), 2199–2204. 10.1073/pnas.1216244110 23341596 PMC3568313

[ece36197-bib-0047] Williams, F. J. , Mills, D. S. , & Guo, K. (2011). Development of a head‐mounted, eye‐tracking system for dogs. Journal of Neuroscience Methods, 194(2), 259–265. 10.1016/j.jneumeth.2010.10.022 21074562

[ece36197-bib-0048] Wilson, G. I. , Holton, M. D. , Walker, J. , Jones, M. W. , Grundy, E. D. , Davies, I. M. , … Wilson, R. P. (2015). A new perspective on how humans assess their surroundings: Derivation of head orientation and its role in 'framing' the environment. PeerJ, 3, e908 10.7717/peerj.908 26157643 PMC4476166

[ece36197-bib-0049] Wilson, G. I. , Norman, B. , Walker, J. , Williams, H. J. , Holton, M. , Clarke, D. , & Wilson, R. P. (2015). In search of rules behind environmental framing; the case of head pitch. Movement Ecology, 3(24). 10.1186/s40462-015-0051-8 PMC4572619 26380712

[ece36197-bib-0050] Wilson, R. P. , Gómez‐Laich, A. , Sala, J.‐E. , Dell'Omo, G. , Holton, M. D. , & Quintana, F. (2017). Long necks enhance and constrain foraging capacity in aquatic vertebrates. Proceedings of the Royal Society B: Biological Sciences, 284(1867), 20172072 10.1098/rspb.2017.2072 PMC5719181 29142117

[ece36197-bib-0051] Wilson, R. P. , Holton, M. D. , di Virgilio, A. , Williams, H. , Shepard, E. L. C. , Lambertucci, S. , … Duarte, C. M. (2018). Give the machine a hand: A Boolean time‐based decision‐tree template for rapidly finding animal behaviours in multisensor data. Methods in Ecology and Evolution, 9(11), 2206–2215. 10.1111/2041-210X.13069

[ece36197-bib-0052] Wilson, R. P. , Holton, M. D. , Walker, J. S. , Shepard, E. L. C. , Scantlebury, D. M. , Wilson, V. L. , … Jones, M. W. (2016). A spherical‐plot solution to linking acceleration metrics with animal performance, state, behaviour and lifestyle. Movement Ecology, 4(1), 1–11. 10.1186/s40462-016-0088-3 27688882 PMC5035456

[ece36197-bib-0053] Wilson, R. P. , Shepard, E. L. C. , & Liebsch, N. (2008). Prying into the intimate details of animal lives: Use of a daily diary on animals. Endangered Species Research, 4(1–2), 123–137. 10.3354/esr00064

[ece36197-bib-0054] Ydesen, K. S. , Wisniewska, D. M. , Hansen, J. D. , Beedholm, K. , Johnson, M. , & Madsen, P. T. (2014). What a jerk: Prey engulfment revealed by high‐rate, super‐cranial accelerometry on a harbour seal (*Phoca* *vitulina*). Journal of Experimental Biology, 217, 2239–2243. 10.1242/jeb.100016 24737765

