# Supplementary material for: An “orientation sphere” visualization for examining animal head movements
Source: Ecol Evol. 2020 Mar 24;10(10):4291–302. doi: 10.1002/ece3.6197 (PMC7246194; doi:10.1002/ece3.6197)
Supplement: Supplementary file 1 — Supplementary Material [file ECE3-10-4291-s001.docx]

Supplementary information

**SI 1** – Details on the tagging procedure.

**Aldabra tortoise**

The study took place on the 26 ha island nature reserve Ile aux Aigrettes, 800 m off the southeast coast of Mauritius (20° 25’ 13.8’’S, 57° 43’ 57’’E), managed by the Mauritian Wildlife Foundation. It is a low-lying coral-limestone island, characterised by sub-tropical seasonal climate with distinctive warm cyclonic seasons, and contains the last remains of the Mauritian dry coastal forest. Tortoises (Aldabra giant tortoises *Aldabrachelys gigantea*) were marked in May 2018, by approaching tortoises from behind and attaching daily diary (‘DD’ Wilson et al. 2008) tags on the carapace and the head using double-sided Velcro tape [with an adhesive/sticky side], without need to capture or handle the animals (which did not show any adverse or escape reactions). The devices were powered with a 3.7V 43mAh battery (1g weight) and stored inside a zip-lock plastic bag attached to the Velcro tape (total weight of tag <5g). Tortoises weighted > 120 kg, thus in all cases the tags were largely below the 3% tag weight limit. The loggers recorded for 1 to 3 hours and were then removed. All tags detached without leaving signs on the skin of the animals.

**Arabian oryx**

The study took place at Mahazat as-Sayd protected area, a large (2244 km^2^) open steppe desert in west-central Saudi Arabia (28°15’ N, 41°40’E) characterised by hot summers, mild winters and low rainfall (mean 96 ± 41 mm *p.a.*) (Ostrowski et al. 2003; Al-Sodany et al. 2011). Arabian oryx were captured during the summer (August 2014) and the winter (February 2015) and after device deployment were released into a (2 x 1 km) fenced enclosure which contained sufficient vegetation for them to eat but no free water or extra food provision.

A daily diary (‘DD’ Wilson et al. 2008) tag was attached to each of a total of 20 individuals using a combination of quick-set epoxy resin (Araldite® Rapid) and cyanoacrylate (Loctite superglue®) glues. This was mounted directly to the oryx’s head between the horns (Fig. 1). Each DD was powered by a 2.1 Ah, 3.6 V lithium-thionyl chloride (Li-SOCl2) cell (Saft LS 17330, mass 14.4 g, dimensions 33.4 × 16.5 mm) were enclosed within the casing, the flat side of which allowed the circuit board to lie adjacent to the body during deployment. An identical tag was attached to the neck using a collar (Fig. 1). All channels from both devices recorded at 40 Hz for a period of 8-10 days.

Fig. 1 – Attachment of DD tags to the head and neck of an Arabian oryx.

**Domestic sheep**

We equipped a Merino sheep with 2 Daily Diaries (http://www.wildbyte-technologies.com/; UK): one in the back of the head and the other attached to a collar with a GPS device (CatLog-B, Perthold Engineering, www.perthold.de; USA). The sheep belonged to a flock of approximately 200 animals that were grazing on an extensive rangeland system in Patagonia, Argentina (Fortín Chacabuco ranch, https://www.nature.org/en-us/about-us/where-we-work/latin-america/argentina/fortin-chacabuco/). The DDs recorded data from a 3-axial accelerometer, a 3-axial magnetometer, external temperature and pressure at 40 Hz. The GPS device was programmed to register location data once per minute. The head DD was glued to the sheep’s head via a temporary epoxy adhesive (Poxipol; Akapol, Argentina) directly to this region after shaving the wool. This device was removed after 15 days by cutting the wool that grew underneath the device.

**Loggerhead turtle**;

A loggerhead sea turtle was equipped with a Daily Diary tag, (DD; 54 x 29 x 22 mm, mass 22 g; Wilson et al., 2008) set to record at 20 Hz per channel. The device was attached to the second central scute of the carapace using a two-part epoxy (Veneziani Subcoat S), pre-mixed in water. During tag attachment the turtle was placed into a plastic box and left for approximately 20 minutes to give the epoxy some time to set.

**SI 2** – The basic code is C++ for creating the O-sphere is as follows;

int datapoints = 0;

              int datapoints_predict = 0;

              for (int i = 0; i < target_events; i++)

              {

                     new_3d_data_array_reference[i + visual_val * events_per_file] = 0;

              }

              for (int i = myViz[visual_val].dataPointStart; i < (myViz[visual_val].dataPointStop + 1); i++)

              {

                     if (((myViz[visual_val].dr_marked_events == 0) | (myViz[visual_val].dr_marked_events == graph_data[i + 87 * target_events]))) datapoints_predict++;

              }

              for (int i = myViz[visual_val].dataPointStart; i < (myViz[visual_val].dataPointStop + 1); i++)

              {

                     if (((myViz[visual_val].dr_marked_events == 0) | (myViz[visual_val].dr_marked_events == graph_data[i + 87 * target_events])))

                     {

                           new_3d_data_array[datapoints * 3 + 0 + 3 * visual_val * events_per_file] = 1;

                           new_3d_data_array[datapoints * 3 + 1 + 3 * visual_val * events_per_file] = 0;

                           new_3d_data_array[datapoints * 3 + 2 + 3 * visual_val * events_per_file] = 0;

                           deg_heading = ((double(graph_data[i + 133 * target_events]) / 4096) / 180) * M_PI;

                           deg_pitch = ((double(graph_data[i + 140 * target_events]) / 500) / 180) * M_PI;

                           if (myViz[visual_val].osphere_zero_pitch) deg_pitch = 0;

                           x = new_3d_data_array[datapoints * 3 + 0 + 3 * visual_val * events_per_file];

                           y = new_3d_data_array[datapoints * 3 + 1 + 3 * visual_val * events_per_file];

                           new_3d_data_array[datapoints * 3 + 0 + 3 * visual_val * events_per_file] = (x * cos(deg_pitch) - y * sin(deg_pitch));

                           new_3d_data_array[datapoints * 3 + 1 + 3 * visual_val * events_per_file] = (x * sin(deg_pitch) + y * cos(deg_pitch));

                           x = new_3d_data_array[datapoints * 3 + 0 + 3 * visual_val * events_per_file];

                           z = new_3d_data_array[datapoints * 3 + 2 + 3 * visual_val * events_per_file];

                           if (!myViz[visual_val].osphere_time_rad)

                           {

                                  new_3d_data_array[datapoints * 3 + 0 + 3 * visual_val * events_per_file] = (x * cos(deg_heading) - z * sin(deg_heading));

                                  new_3d_data_array[datapoints * 3 + 2 + 3 * visual_val * events_per_file] = (x * sin(deg_heading) + z * cos(deg_heading));

                           }

                           else

                           {

                                  new_3d_data_array[datapoints * 3 + 0 + 3 * visual_val * events_per_file] = (x * cos(deg_heading) - z * sin(deg_heading)) * (double(datapoints) / double(datapoints_predict));

                                  new_3d_data_array[datapoints * 3 + 2 + 3 * visual_val * events_per_file] = (x * sin(deg_heading) + z * cos(deg_heading)) * (double(datapoints) / double(datapoints_predict));

                           }

                           new_3d_data_colour_array[datapoints * 4 + 0 + 4 * visual_val * events_per_file] = 1;

                           new_3d_data_colour_array[datapoints * 4 + 1 + 4 * visual_val * events_per_file] = 1;

                           new_3d_data_colour_array[datapoints * 4 + 2 + 4 * visual_val * events_per_file] = 1;

                           new_3d_data_colour_array[datapoints * 4 + 3 + 4 * visual_val * events_per_file] = 1;

                           if (datapoints > 0)

                           {

                                  graph_data[i + 62 * target_events] = graph_data[i + 140 * target_events] - graph_data[(i - 1) + 140 * target_events]; // diff pitch

                                  if (graph_data[i + 62 * target_events] < 0 & opengl_osphere_abs_diff_hdg_pitch_cb_val) graph_data[i + 62 * target_events] = -graph_data[i + 62 * target_events];

                                  graph_data[i + 63 * target_events] = graph_data[i + 133 * target_events] - graph_data[(i - 1) + 133 * target_events]; // diff heading

                                  if (graph_data[i + 63 * target_events] < 0) graph_data[i + 63 * target_events] = -graph_data[i + 63 * target_events];

                                  if (graph_data[i + 63 * target_events] > 737280) graph_data[i + 63 * target_events] = 1474560 - graph_data[i + 63 * target_events];

                                  if (graph_data[i + 63 * target_events] < 0) graph_data[i + 63 * target_events] = -graph_data[i + 63 * target_events];

                                  a = (new_3d_data_array[datapoints * 3 + 0 + 3 * visual_val * events_per_file] * new_3d_data_array[(datapoints - 1) * 3 + 0 + 3 * visual_val * events_per_file] + \

                                         new_3d_data_array[datapoints * 3 + 1 + 3 * visual_val * events_per_file] * new_3d_data_array[(datapoints - 1) * 3 + 1 + 3 * visual_val * events_per_file] + \

                                         new_3d_data_array[datapoints * 3 + 2 + 3 * visual_val * events_per_file] * new_3d_data_array[(datapoints - 2) * 3 + 2 + 3 * visual_val * events_per_file]);

                                  b = sqrt(new_3d_data_array[datapoints * 3 + 0 + 3 * visual_val * events_per_file] * new_3d_data_array[datapoints * 3 + 0 + 3 * visual_val * events_per_file] + \

                                         new_3d_data_array[datapoints * 3 + 1 + 3 * visual_val * events_per_file] * new_3d_data_array[datapoints * 3 + 1 + 3 * visual_val * events_per_file] + \

                                         new_3d_data_array[datapoints * 3 + 2 + 3 * visual_val * events_per_file] * new_3d_data_array[datapoints * 3 + 2 + 3 * visual_val * events_per_file]);

                                  c = sqrt(new_3d_data_array[(datapoints - 1) * 3 + 0 + 3 * visual_val * events_per_file] * new_3d_data_array[(datapoints - 1) * 3 + 0 + 3 * visual_val * events_per_file] + \

                                         new_3d_data_array[(datapoints - 1) * 3 + 1 + 3 * visual_val * events_per_file] * new_3d_data_array[(datapoints - 1) * 3 + 1 + 3 * visual_val * events_per_file] + \

                                         new_3d_data_array[(datapoints - 1) * 3 + 2 + 3 * visual_val * events_per_file] * new_3d_data_array[(datapoints - 1) * 3 + 2 + 3 * visual_val * events_per_file]);

                                  graph_data[i + 64 * target_events] = 4096 * a / (b * c); // diff o-sphere

                                  if (graph_data[i + 64 * target_events] < 0 & opengl_osphere_abs_diff_hdg_pitch_cb_val) graph_data[i + 64 * target_events] = -graph_data[i + 64 * target_events];

                           }

                           new_3d_data_array_reference[datapoints + visual_val * events_per_file] = (i - myViz[visual_val].dataPointStart);

                           datapoints++;

                     }

              }

**Supplementary video**.

Animation showing an expanding O-sphere of the head behaviour of an Arabian oryx (cf. Fig. 7) representing a total of 100 s. To facilitate interpretation, the line has been coloured by pitch, with greens showing pitch-down. At the outset, the arrow starts at time = 0, shows the planer view indicating the head heading. The arrow then traces the time line and the change in head headings until the point of maximum radius. The O-sphere is then rotated so that the pitch aspect of the behaviours can be seen before the sphere is rotated back to its original position.

**References**

Ostrowski, S., Bedin, E., Lenaina, D.M., & Abuzinadaa, A.H. (1998). Ten years of Arabian oryx conservation breeding in Saudi Arabia - achievements and regional perspectives. *Oryx*, **32**, 209-222.

 Al-Sodany, Y.M., Mosallam, H.A., & Bazaid, S.A. (2011). Vegetation analysis of Mahazat Al-Sayd Protected Area: The second Largest Fenced Nature Reserve in the World. *World Appl Sci J*, **15**, 1144-1156.
